# A Geometrical Account to Explain the Fat-Face Illusion

**DOI:** 10.1177/2041669520981094

**Published:** 2020-12-22

**Authors:** Amit Rawal, Philip Tseng

**Affiliations:** Graduate Institute of Mind, Brain, & Consciousness, Taipei Medical University, Taipei City; Graduate Institute of Mind, Brain, & Consciousness, Taipei Medical University, Taipei City; Brain and Consciousness Research Center, Shuang Ho Hospital, Taipei Medical University, Taipei City; Psychiatric Research Center, Wan Fang Hospital, Taipei Medical University, Taipei City

**Keywords:** face perception, frames of reference, shape, shapes/objects

## Abstract

Investigations of the “fat-face” illusion have unanimously agreed that the illusion is face-specific. Here, we offer several manipulations to highlight that the fat-face illusion is not restricted to the bottom image, isn’t a property of internal features, facial contour/texture, and in general isn’t even specific to faces. We propose the axis of horizontal asymmetry account to contextualize fat-face illusion as a geometry-led illusion.

Recent work on between-face comparisons for difference in apparent size (“fatness”) has surfaced since Thompson’s (2010) “fat-face-thin illusion,” where a face appears thinner when inverted than upright. Thompson & Wilson (2012) suggested the configuration of internal facial features (eyes/nose/mouth) to be crucial, as the disrupted holistic processing in inverted faces would lead to misjudgments about its external features (contour/geometry). Importantly, Thompson concluded that the external features themselves do not contribute to the “fat-face-thin illusion,” based on the observation that faces without internal features appear equally fat.

Subsequently, Sun et al. (2012) reported a novel fat-face illusion that does not require inversion. Two identical upright faces, when stacked vertically, make the lower face appear 4% “fatter.” They compared these to inverted faces and clocks and found the effect restricted to upright faces. Importantly, line drawings of faces without facial features, or intact facial features without face contour, were both sufficient in inducing the illusion (albeit weakly). The authors concluded that face contour and internal features may, independently, activate a face schema, sufficing to result in basic-level facial processing necessary for the illusion (Sun et al., 2013).

Zhong et al. (2012) explored the effect of facial outlines using oval-, trapezoid-, and hexagon-outlined faces. They found the illusion present for upright but not inverted ovals, for upright and also inverted trapezoids (with direction of illusion reversed for inverted trapezoids), and no effect for hexagon-outlined faces. These results are confusing because the illusion for trapezoid faces depended entirely on orientation, yet oval faces only had an effect for upright faces. The authors speculated that the absence of illusion in hexagon faces was due to the lack of visual expertise for hexagonal faces.

Recently, Tomonaga (2015) mentioned insights by Kitaoka (2006), that the fat-face illusion may be the result of a comparison between the lower (narrow) part of the upper face and the upper (wider) part of the lower face, following a Jastrow–Esque comparison between the two faces, rendering the lower face to appear “fatter.” Here, we expand on this idea and present the axis of horizontal asymmetry (AHA) account that aims to explain the fat-face illusion in terms of the geometry of the stimuli, where the compared stimulus may or may not be a face.

In Figure 1, two separate faces are shown in different alignments, with corresponding changes in the direction of illusion. In all alignments, one face’s chin (i.e., narrow) points to another face/head (i.e., wider). The face being pointed at appears larger.

**Figure 1.**
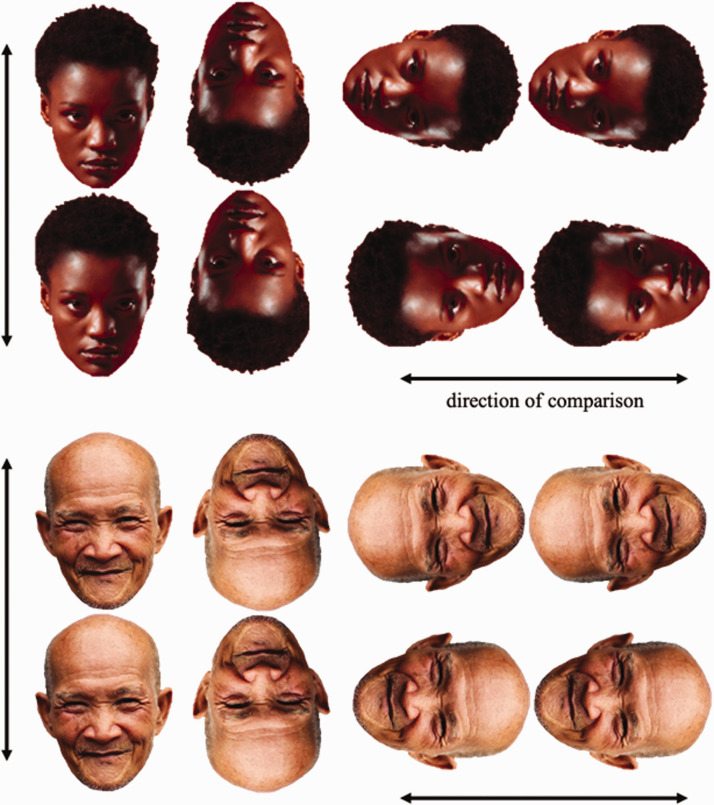
Fat-face illusion with upright, inverted, and side-aligned faces.

Following face comparisons in upright, inverted, and rotated orientations in Figure 1, we attempt to create this illusion with sideways faces, where the face pointing at the back of the other face may appear less wide. Similar to Figure 1, the effect may emerge in a case where the narrower part (mouth/nose) meets the other’s wider back.

It was proposed by Sun et al. (2013) that the fat-face illusion operates via a top-down mechanism that requires the activation of facial schema to facilitate the illusion. The AHA account we propose would predict that as long as the geometry is preserved, the illusion should persist. Therefore, we hypothesize that the illusion should be present in Figure 3, with scrubbed facial features (middle) but also with a mere monochromatic head mask (left).

To test whether this illusion is face-specific, clocks and buildings comparisons are shown in Figure 4. We predict that the canonical (upright) orientation of an object has little to do with the fat “face” illusion. The first (circular) pair of clocks does not showcase the illusion (Sun et al., 2012) because they are perfectly circular. However, as geometrical asymmetry is introduced, the illusion becomes apparent in the trapezoid clock and building. The direction of the illusion is predicted to be consistent with the geometrical account (i.e., pointy = thinner/smaller; pointed = fatter/larger).

So far, the AHA account posits that the horizontal asymmetry of the faces is driving the previously reported findings. If so, then an existing effect should be strengthened upon manipulation of this feature. This is attempted in Figure 5 by shaving the neck and chin.

We further hypothesize that even the direction of the fat-face illusion should be reversed if the geometry demands it. This is demonstrated in Figure 6 using a face with narrower top and wider bottom. We predict a direction reversal in the illusion, which will further highlight the influence of geometry.

To validate our observations shown in the figures (Figures 1 [Fig fig2-2041669520981094][Fig fig3-2041669520981094][Fig fig4-2041669520981094][Fig fig5-2041669520981094]to 6), we conducted a survey via Google forms (*N* = 61, 15 males, mean age = 26, age range = 19 to 42). For each question, respondents were shown one pair of faces/objects and were instructed to make a binary choice response by selecting the one that appeared relatively larger/fatter. To keep the size of the images relatively similar across respondents, the instructions mandated the use of large screens (not smartphones/tablets). The protocol of this survey was approved by the Joint Institutional Review Board of Taipei Medical University.

The stimuli for survey consisted of 34 items, which included the two faces shown in Figure 1, in upright, inverted, and rotated (right-pointing/left-pointing) orientations (8 comparisons), the two faces shown in Figure 2, presented in both right-facing and left-facing orientations (4 comparisons), the two faces from Figure 3, with either the facial features erased or with a mask of the whole head (8 comparisons), the round and trapezoid clocks, and the trapezoid building from Figure 4 (6 comparisons), the original and modified face in Figure 5 (2 comparisons), and the face in Figure 6 in upright and inverted orientation (2 comparisons).

**Figure 2. fig2-2041669520981094:**
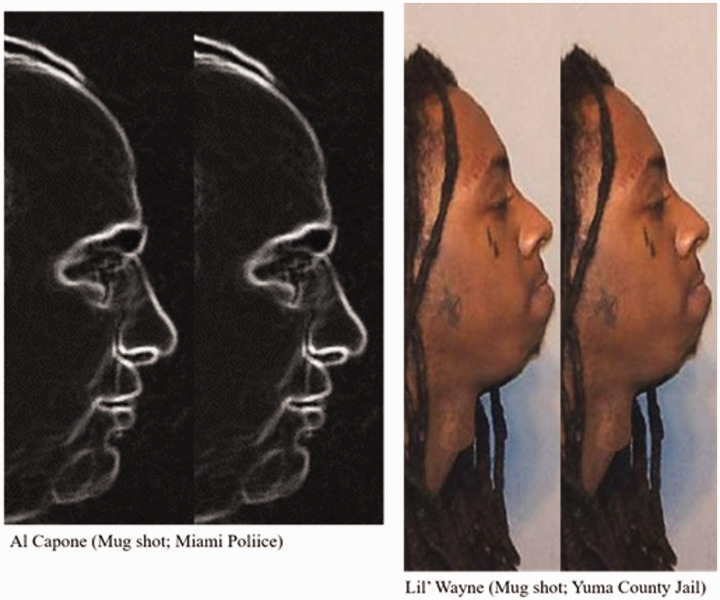
A variant of face comparisons where sideways faces may result in the fat-face illusion.

**Figure 3. fig3-2041669520981094:**
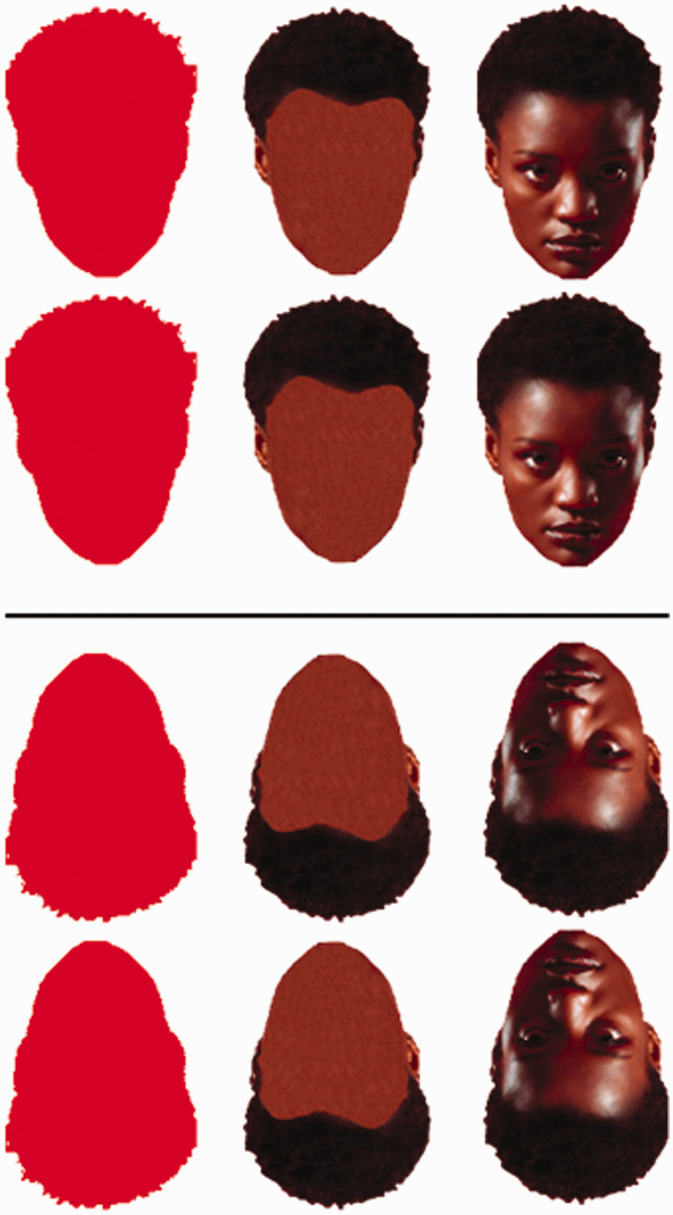
Faces without internal features, and head masks, may also induce the illusion.

**Figure 4. fig4-2041669520981094:**
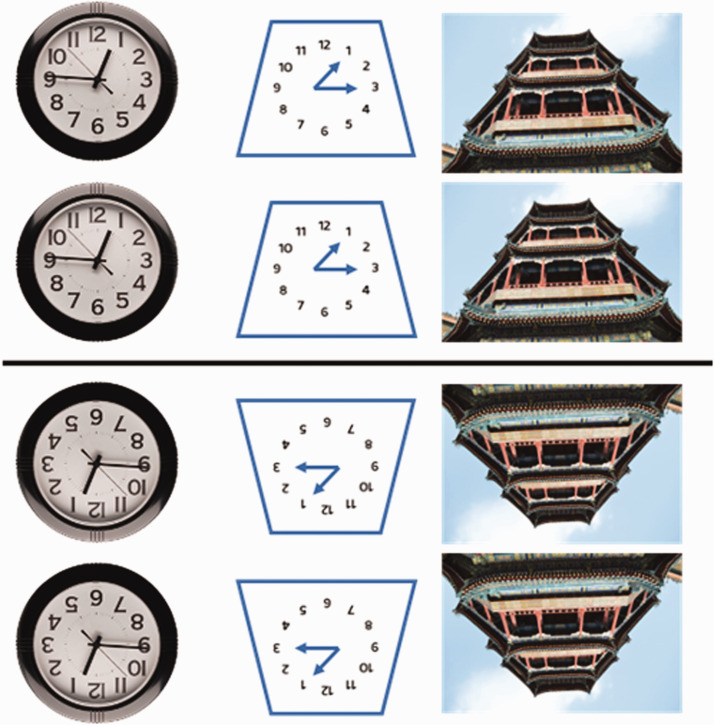
Nonface stimuli, given a trapezoid-enough shape, can also induce illusion. Circular and hexagonal shapes are unsuitable controls due to their perfect horizontal symmetry.

**Figure 5. fig5-2041669520981094:**
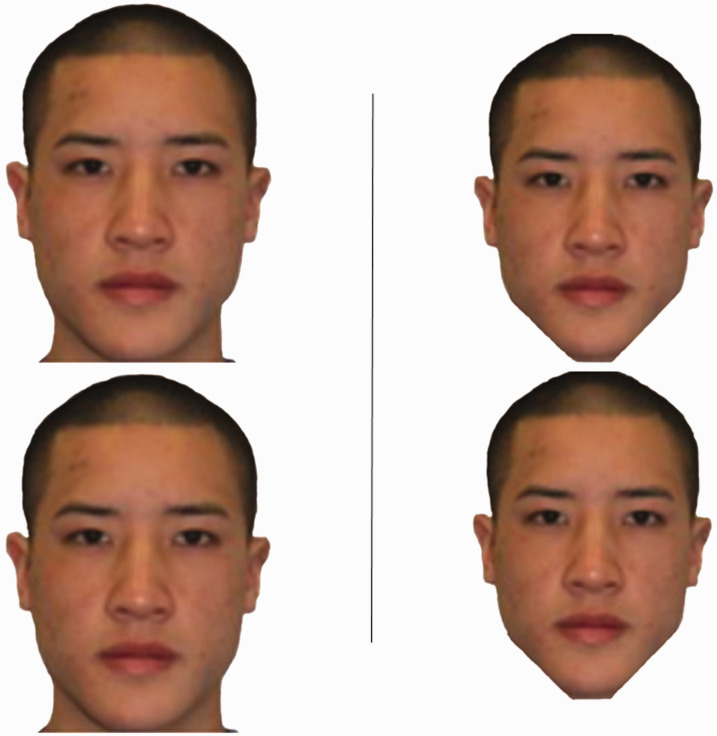
Existing effects might seem modified/amplified if the geometry is changed such that the lower region of face is pointier and top region is flatter/wider. Original image (left) adapted from Sun et al. (2013).

**Figure 6.**
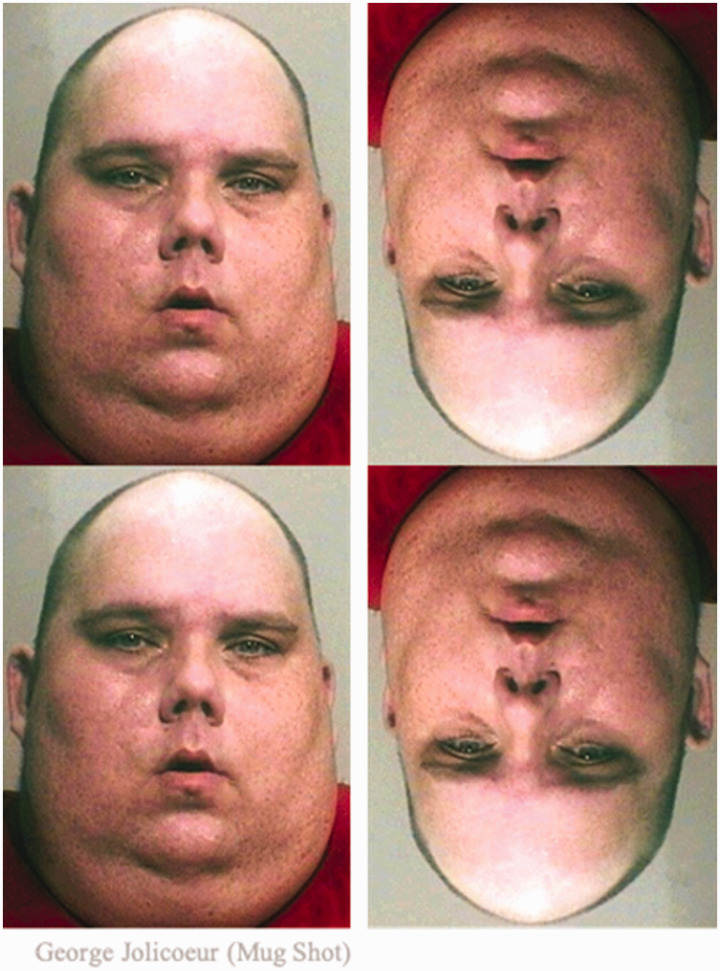
Face with opposite to common head shape may show a reversed fat-face illusion.

In addition, to look for the possible effect of distance between the two stimuli, we took the female face from Figure 1 and presented it upright at four more distances, which were twice as distant as the original, and then again double the distance for each of the three subsequent comparisons (four comparisons).

The direction of the illusions was predicted by the AHA account, mentioned previously. All of the 34 items (comparisons) were presented in three different randomized orders, and 20, 20, and 21 participants, respectively, responded to these orders. The number of responses in the hypothesized direction, from these three orders, is shown in Table 1. Responses from all participants (*N* = 61) were pooled and compared against the hypothesized direction, using one-sample binomial tests, against a chance level of 0.5 (50%) of picking either direction (up/down or left/right). To quantify the effect size for these tests, we used Cohen’s *g* (Cohen, 1988), which is equal to [observed proportion—expected proportion (null)]. These comparisons are depicted in Figure 7, and effect size values are shown in Table 2. For a two-tailed binomial test involving 61 participants, a statistically significant outcome would be one that results in a Cohen’s *g* value of either at or above (0.139) or at or below (–0.139).

**Table 1.** Number of Successful Illusions in the Predicted Direction, per Comparison, for Each Order of Stimulus Presentation.

Comparison	1	2	3	4	5	6	7	8	9	10	11	12	13	14	15	16	17
Order #1	14	17	17	19	14	13	13	14	18	15	14	12	13	13	11	11	16
Order #2	17	17	18	18	12	16	11	15	19	17	13	12	16	11	14	13	11
Order #3	18	18	21	20	13	15	19	20	19	20	14	16	13	16	15	13	18
Comparison	18	19	20	21	22	23	24	25	26	27	28	29	30	31	32	33	34
Order #1	14	15	13	15	13	17	9	9	13	19	19	17	18	14	14	11	14
Order #2	12	16	14	17	15	15	5	10	11	20	20	18	19	16	16	17	12
Order #3	18	16	14	20	14	18	9	11	10	21	21	19	18	19	16	18	17

*Note*. The three orders contain responses from 20, 20, and 21 participants, respectively.

**Figure 7.**
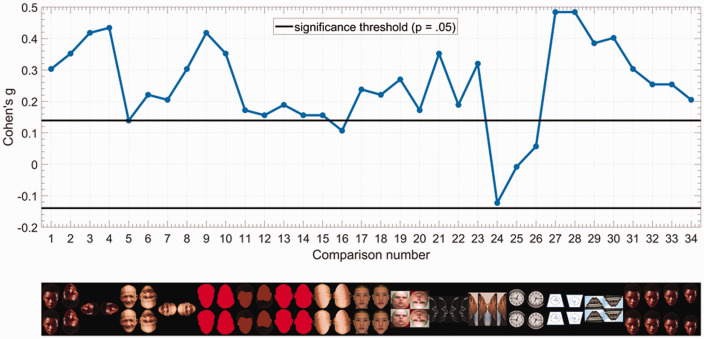
Summary of results: The upper plot shows the effect size, Cohen’s *g*, of a one-sample binomial test (against 50%), for each comparison. The greater the distance from zero, the stronger the effect. The direction of the comparison was hypothesized by the AHA account (i.e., the stimulus that points to the other stimulus will look smaller/thinner, and the pointed stimulus will look larger/fatter). The black lines mark the *p* = .05 significance threshold for the two-tailed tests. Statistically significant comparisons are ones with Cohen’s *g* values at or above 0.139 (upper line) or at or below –0.139 (lower line). The montage at the bottom shows the images corresponding to the comparisons (*x* axis) of the upper figure. (Note: For Comparisons 3, 4, 7, and 8, only one rotated face [out of 2] is shown due to space constraints. Please refer to Figure 1 for the actual stimulus images.) The only comparison that went against our predicted direction is Comparison 24. Null results in Comparisons 25 and 26 are also consistent and predicted by the AHA account.

**Table 2.** Cohen’s *g* Values for the Comparisons Depicted in Figure 7.

Comparison	1	2	3	4	5	6	7	8	9	10	11	12
Cohen’s *g*	0.3	0.35	0.41	0.43	0.13	0.22	0.2	0.30	0.41	0.35	0.17	0.15

Comparison	13	14	15	16	17	18	19	20	21	22	23	24
Cohen’s *g*	0.18	0.15	0.15	0.1	0.23	0.22	0.27	0.17	0.35	0.18	0.32	–0.12

Comparison	25	26	27	28	29	30	31	32	33	34		
Cohen’s *g*	–0.008	0.05	0.48	0.48	0.38	0.4	0.3	0.25	0.25	0.2		

From the results (Figure 7 and Table 2), we can see that for most comparisons, an effect is evident in the predicted direction. The exception is Comparison 24 (*g* = –0.12), where out of two leftward facing faces, majority of the responses indicated that the right face was larger, while we predicted otherwise. Comparison 23 with rightward-facing faces also had majority of the responses indicating the right face (predicted direction) to be larger (*g* = 0.31). Because the two comparisons used images that are mirror images of one another, it might be the case that there is a rightward bias, but it seems particular to this stimulus because Comparisons 21 and 22, also mirrored-images, both received responses in the predicted directions (*g* = 0.35 and *g* = 0.18, respectively). Yet, due to the inconsistency, we leave this effect open to interpretation. The rest of the comparisons, which are either vertically or horizontally stacked faces/clocks/buildings, showed a consistent effect in the direction predicted by AHA. Furthermore, Comparisons 25 and 26, where AHA predicts an absence of illusion (see Firestone & Scholl, 2014) with upright and inverted round clocks, indeed resulted in near-zero Cohen’s *g* of (–0.008) and (0.05), respectively. In contrast, both the upright and inverted trapezoid clocks in Comparisons 27 and 28 resulted in an effect of (0.48), and the upright and inverted trapezoid buildings in Comparisons 29 and 30 (Figure 4) resulted in effects of (0.38) and (0.4), respectively. This makes it evident how this illusion is not specific to faces and not caused by the relative “fatness” of one of the stimuli, given that clocks and buildings may appear larger but not fatter, per se.

In addition, contrary to previous studies, inverted-face comparisons also showed a (*g* > 0.1) effect, whether facial features were present or not (Comparisons 2, 6, 10, 12, 14, and 16), but in the opposite direction (upper image appearing larger). Even faces rotated 90 degrees in the clockwise and counterclockwise direction showed (*g* > 0.2) effects, where the face pointing the chin towards the other face appeared smaller (Comparisons 3, 4, 7, and 8). And interestingly, a face with an opposite-to-canonical geometry, where the upper part of the face is narrower than the lower part of the face, demonstrated a reversal in the direction previously reported for faces, with the upper face appearing larger (*g* = 0.27) for the upright orientation, and the lower face appearing larger (*g* = 0.17) in the inverted orientation (Comparisons 19 and 20; Figure 6). This further highlights the influence of (facial) geometry for this type of illusion and suggests that previous observations of the lower face appearing fatter were likely driven by the canonical face geometry. For the faces shown in Figure 5, our attempt at enhancing the strength of the illusion by sharpening the lower part of the face did not result in a stronger effect (0.23 vs. 0.22). It seems to be the case that the illusion will not become more prevalent upon enhancing the facial geometry, but it could be that the magnitude of the illusion will be strengthened, for those who do experience the illusion. Future studies could explore such differences in the magnitude of this illusion.

Finally, faces shown at incremental distance apart also showed an illusion, where the effect seems to slowly decline with distance between the faces, with the original effect (Comparison 1) being (0.3), and the effects for Comparisons 31 to 34 being (0.3), (0.25), (0.25), and (0.2), respectively. This would be consistent with Kitaoka’s (2006) suggestion that the fat-face illusion may be the result of a comparison between the lower (narrow) part of the upper face and the upper (wider) part of the lower face. This potential effect of distance also hints at a possible role of visual attention, or even local featural comparisons at the foveal level, though we can only speculate at this moment as the levels of attentional engagement or viewing distance and visual angles cannot be properly controlled in an online study. Nevertheless, this effect of distance opens up new possibilities for future studies that can potentially reveal the mechanisms underlying the fat-face illusion and its AHA principles.

Following these results, we revisit previous studies to highlight how previous cases where no illusion was observed also happened to have a symmetric geometry (Figure 8). The only exception to this account is highlighted in red, where an oval face was found to exhibit the illusion (Zhong et al., 2012).

**Figure 8.**
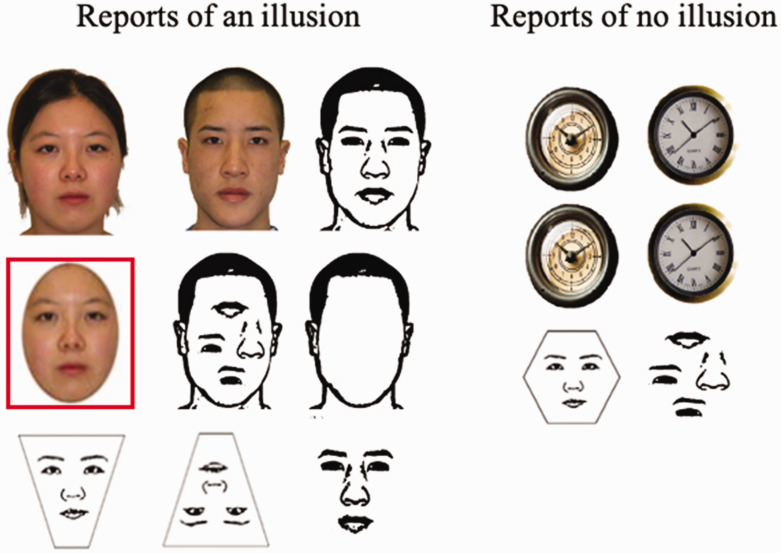
List of the presence and absence of the fat-face illusion (Sun et al., 2012, 2013; Zhong et al., 2012).

In conclusion, we demonstrate how upright, inverted, horizontally aligned sideways faces (perhaps) and also face masks and nonfaces (clock, buildings) may exhibit a “fat-face” illusion. We think the AHA account can accommodate all variants of the fat-face illusion, though other factors such as internal facial features may exert an additive effect and possibly influence the perception of external features (Thompson & Wilson, 2012; Zhong et al., 2012).
